# Enhanced Production of β-Caryophyllene by Farnesyl Diphosphate Precursor-Treated Callus and Hairy Root Cultures of *Artemisia vulgaris* L.

**DOI:** 10.3389/fpls.2021.634178

**Published:** 2021-03-30

**Authors:** Sundararajan Balasubramani, B. D. Ranjitha Kumari, Anil Kumar Moola, D. Sathish, G. Prem Kumar, S. Srimurali, R. Babu Rajendran

**Affiliations:** ^1^College of Horticulture and Landscape Architecture, Southwest University, Chongqing, China; ^2^Department of Botany, Bharathidasan University, Tiruchirappalli, India; ^3^Aditya Degree and P.G. College, Kakinada, India; ^4^Department of Biotechnology, Bharathidasan University, Tiruchirappalli, India; ^5^China-USA Citrus Huanglongbing Joint Laboratory, National Navel Orange Engineering Research Center, Gannan Normal University, Ganzhou, China; ^6^ICMR-National Institute of Nutrition, Hyderabad, India; ^7^Department of Environmental Biotechnology, Bharathidasan University, Tiruchirappalli, India

**Keywords:** *Artemisia vulgaris* L., farnesyl diphosphate, gas chromatography–mass spectrometry, precursor treatment, essential oil

## Abstract

*Artemisia vulgaris* L. produces a wide range of valuable secondary metabolites. The aim of the present study is to determine the effects of various concentrations of farnesyl diphosphate (FDP) on β-caryophyllene content in both callus and hairy root (HR) cultures regeneration from leaf explants of *A. vulgaris* L. Murashige and Skoog (MS) medium supplemented with various concentrations of 2,4-dichlorophenoxyacetic acid (2,4D; 4–13 μM), α-naphthaleneacetic acid (NAA; 5–16 μM), and FDP (1 and 3 μM) was used for callus induction and HR regeneration from leaf explants of *A. vulgaris* L. In this study, precursor-treated (2,4D 13.5 μM + FDP 3 μM) callus displayed the highest biomass fresh weight (FW)/dry weight (DW): 46/25 g, followed by NAA 10.7 μM + FDP 3 μM with FW/DW: 50/28 g. Two different *Agrobacterium rhizogenes* strains (A_4_ and R_1000_) were evaluated for HR induction. The biomass of HRs induced using half-strength MS + B_5_ vitamins with 3 μM FDP was FW/DW: 40/20 g and FW/DW: 41/19 g, respectively. To determine β-caryophyllene accumulation, we have isolated the essential oil from FDP-treated calli and HRs and quantified β-caryophyllene using gas chromatography–mass spectrometry (GC–MS). The highest production of β-caryophyllene was noticed in HR cultures induced using A_4_ and R_1000_ strains on half-strength MS medium containing 3 μM FDP, which produced 2.92 and 2.80 mg/ml β-caryophyllene, respectively. The optimized protocol can be used commercially by scaling up the production of a β-caryophyllene compound in a short span of time.

## Introduction

*Artemisia vulgaris* L. is an important medicinal herb that belongs to the family Asteraceae. These plants are found in several temperate countries in Europe, Asia, Northern Africa, Alaska, and North America. *A. vulgaris* L. has been widely used as a traditional medicine for treating a number of diseases, such as neonatal jaundice (Fok, 2002), gastric ulcers (Repetto and Llesuy, 2002), and hepatitis, as well as an analgesic agent in acupuncture therapy (Yoshikawa et al., 1996). Traditionally, it has been effective as antiviral (Tan et al., 1999), insect-repellant, fumigant (Wang et al., 2006), insecticide (Chantraine et al., 1998), antibacterial, anti-inflammatory (Tigno et al., 2000), sedative, and flavoring and perfumery agents (Da Silva, 2004). A phytochemical study has identified more than 20 flavonoids in *A. vulgaris* L. extracts (Lee, 1998). Williams et al. (2012) have identified 22 significant chemical components in the essential oil of *A. vulgaris* L.—the major components being β-caryophyllene, α-zingiberene, borneol, and α-curcumin. It also has been found that some species of the plant kingdom produce sesquiterpene β-caryophyllene (De Moraes et al., 2001).

**FIGURE 1:**
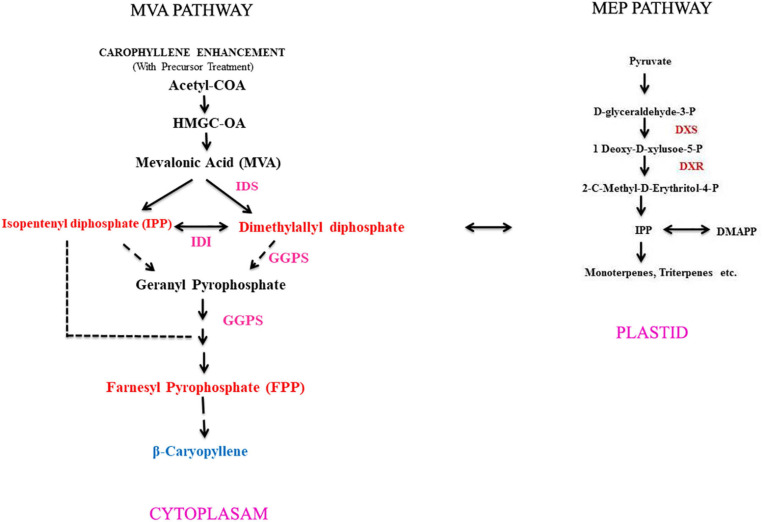
An outline for β-caryophyllene compound biosynthesis pathway operating through *in vitro* method of *A. vulgaris* plant. IDS, isopentenyl diphosphate synthase; IDI, isopentenyl diphosphate isomerase; GGPP, geranylgeranyl diphosphate; DXS, 1-deoxy-D-xylulose-5-phosphate synthase; DXR, 1-deoxy-D-xylulose-5-phosphate reductoisomerase.

Caryophyllene is present in *Zingiber nimmonii* rhizome essential oil that helps curing fungal and bacterial infection (Sabulal et al., 2006). Gertsch et al. (2008) have reported about β-caryophyllene compound that was shown to interact with the binding site cannabinoid receptor type 2 (CB2), but not to type 1 (CB1). This chemical compound is energetically anti-inflammatory and has anesthetic properties. Amiel et al. (2012) reported that the essential oil (β-caryophyllene) of *Commiphora gileadensis* (Balm of Gilead, mentioned in the Bible) has an anti-proliferative property against tumor cell lines. They also found that β-caryophyllene caused a strong induction of apoptosis accompanied by DNA ladder formation and caspase-3 catalytic activity in tumor cell lines. However, the plant sesquiterpenes biosynthesis pathway is considered as an isopentenyl diphosphate (IPP), which is the universal biological precursor of all isoprenoids (basic C_5_ isoprene unit). It can be obtained through either mevalonate (MVA) or 2-methylerythritol 4-phosphate (MEP) pathways (Figure 1). Moreover, it has been well established that the acetyl-coenzyme A (acetyl-CoA) pathway in plants is located in the cytosol, whereas the MEP pathway is targeted to plastids inside (Croteau et al., 2000). There are studies that indicate cross-talk and possible IPP exchange between the other two paths through the cytosol and plastids (Adam and Zapp, 1998; Laule et al., 2003). There were also two independent pathways leading to IPP: the mevalonate pathway (MVA) originating from acetyl-CoA and the pyruvate-derived mevalonate-independent pathway (MEP) (Croteau et al., 2000). IPP instead contributes to the other terpenoids with its isomer dimethylallyl pyrophosphate (DMAPP). The MVA isoprenoid pathway is used by eukaryotes apart from plants, although prokaryotes with many exemptions use the MEP pathway to create IPP and DMAPP separately (Lichtenthaler, 1999; Boucher and Doolittle, 2000). Isopentenyl phosphate kinases (IPKs) generated by plant genomes are derived isopentenyl phosphate (IP) *via* ATP-dependent phosphorylation, forming the primary metabolite IPP commonly used for isoprenoid/terpenoid biosynthesis (Henry et al., 2018). The objective of the present study is to enhance β-caryophyllene content in *in vitro* callus and hairy root (HR) cultures and to isolate the essential oil from both calli and HRs in order to understand the metabolic profile of β-caryophyllene using gas chromatography–mass spectrometry (GC–MS).

## Materials and Methods

### Callus Induction

Seeds of *A. vulgaris* L. were collected from Johnny’s Selected Seeds, Winslow, ME, United States. The botanical identity of *A. vulgaris* was confirmed by comparing them with respect to the reference standards at Johnny’s Selected Seeds. After that, 35-day leaf explants excised from old seedlings were inoculated into Murashige and Skoog (MS) medium (Murashige and Skoog, 1962) supplemented with 2,4-dichlorophenoxyacetic acid (2,4D) and α-naphthaleneacetic acid (NAA) (Sigma-Aldrich, Inc., St. Louis, MO, United States) for callus induction. Callus was initiated by inoculating leaf explants from a 35-day-old plant into MS medium supplemented with various concentrations and combinations of 2,4D (4.5–13 μM) and NAA (5–16 μM), with farnesyl diphosphate (FDP; 1 and 3 μM) (Sigma-Aldrich, Inc., St. Louis, MO, United States) as a precursor in the experiment, followed by a previous report by Sundararajan and Kumari (2015). Fresh weight (FW) and dry weight (DW) were recorded in precursor-treated callus after 60 days (Sivanandhan et al., 2014).

### HR Culture

#### Bacterial Culture Preparation

From the overnight glycerol stock, 500 μl was taken and transferred to the Luria–Bertani (LB) medium, and the same was subcultured thrice to obtain active culture. The Gram-negative soil bacteria *Agrobacterium rhizogenes* (A_4_ and R_1000_) transferred to the medium were cultured overnight at 25°C in an orbital shaker at 180 rpm. The bacterial cells were harvested by centrifugation at 5,000 rpm for 15 min at 25°C. The pellet-containing bacterial cells were re-suspended in liquid MS medium, and the final optical density (OD) was adjusted to 0.6.

#### Induction and Establishment of HR Culture

In aseptic condition, leaf explants derived from 45-day-old plants were wounded with a sterile scalpel, transferred into the *A. rhizogenes* infection medium, and incubated for 10 min at 25°C. After infection, the explants were dried on a sterile filter paper and placed on semisolid 1/2 MS medium supplemented with 3% sucrose and supplemented separately with 50, 100, and 150 μM of acetosyringone (AS; Sigma, United States). AS was filter-sterilized by using a 0.2-μm syringe filter 109 (Pall Corp., United States) and added to the cooled autoclaved medium. After cocultivation, explants were washed with liquid 1/2 MS containing 500 mg/L cefotaxime, followed by sterile distilled water. The explants were then inoculated on 1/2 MS medium supplemented with 500 mg/L cefotaxime and kept under dark condition for HR induction. HRs of more than 1–2 cm length were excised from the explants and transferred to 30 ml 1/2 MS, 1/2 MS + B_5_ vitamins, 1/2 MS + FDP 1 μM, 1/2 MS + FDP 3 μM, 1/2 MS + B_5_ vitamins + FDP 1 μM, and 1/2 MS + B_5_ vitamins + FDP 3 μM supplemented with 3% sucrose with 500 mg/L cefotaxime until the residual bacteria are killed completely. The experiment was run thrice with 25 explants for each experiment.

#### Determination of the Growth Index of the HR Cultures

Experiments were conducted to evaluate the growth rate of the hair roots grown in 1/2 MS, 1/2 MS + B_5_ vitamins, 1/2 MS + FDP 1 μM, 1/2 MS + FDP 3 μM, 1/2 MS + B_5_ vitamins + FDP 1 μM, and 1/2 MS + B_5_ vitamins + FDP 3 μM. The growth index (GI) of all the four lines was measured initially and inoculated with 20 mg of FW bacteria-free HRs (3 weeks old). It was transferred into a 30 ml liquid medium in a 250 ml conical flask and maintained on a rotary shaker at 120 rpm in a dark room for 12 weeks. The *in vitro* grown HRs were subcultured in a fresh medium for 6 weeks to stimulate further growth of the hairs. Each treatment consisted of three replicates, and each replicate contained 25 explants per treatment. The GI was determined by using the following formula (Ashraf et al., 2015):

$$\mathrm{Growth}\,\mathrm{index}\,\left(\text{GI}\right)=\frac{\mathrm{Final}\,\mathrm{biomass}\,\mathrm{weight}-\mathrm{Initial}\,\mathrm{biomass}\,\mathrm{weight}}{\mathrm{Initial}\,\mathrm{biomass}\,\mathrm{weight}}.$$

FW and DW of HRs were recorded after 12 weeks, according to the method of Sivanandhan et al. (2014).

### Polymerase Chain Reaction Analysis

Integration of the T-DNA, which is responsible for HR formation, was confirmed by polymerase chain reaction (PCR) analysis using *rolB* gene and *rolC* gene-specific primers (Cho et al., 1998). Genomic DNA was isolated from both the control non-transformed roots and the transformed HR cultures by using the cetyltrimethylammonium bromide (CTAB) method (Khanuja et al., 1999). The following primers were used for PCR analysis: for *rolB* gene, forward primers—5-ATG GAT CCC AAA TTG CTA TTC CCC CAC GA-3 and reverse primers—5-TTA GGC TTC TTT CAT TCG GTT TAC TGC AGC-3 and for *rolC* gene, forward primers—5-ATG GCT GAA GAC GAC CTG TGT T-3 and reverse primers—5-TTA GCC GAT TGC AAA CTT GCA C-3. The PCR was carried out by amplifying with an initial denaturation at 94°C for 5 min, followed by 35 cycles of denaturation at 94°C for 1 min, annealing at 55°C for 1 min *rolB* and *rolC*, and extension at 72°C for 1 min with a final extension of 72°C for 10 min using an Applied Biosystems Veriti Thermal Cycler (Thermo Fisher Scientific, United States). The amplified products were analyzed by electrophoresing 1.4% (*w/v*) of agarose gel along with 100 bp DNA marker (New England Biolabs, United Kingdom) staining with ethidium bromide.

### Essential Oil Isolation and Quantification of β-Caryophyllene Content in Precursor-Treated Callus and HR by GC–MS Analysis

#### Isolation of Essential Oil

Calli and HRs (10 g each) were subjected to hydrodistillation for 4 h at 60°C using a Clevenger-type apparatus. The obtained essential oil was dried over anhydrous sodium sulfate (Na_2_SO_4_). The oil was then filtered and stored at 4°C for further experiments.

#### GC–MS Analysis

The extracted sample was cleaned by passing it through a silica gel column (glass column) containing glass wool, silica gel 60 (230–400 mesh; Merck, United States), and anhydrous Na_2_SO_4_, which helps to remove high-molecular-weight polar substances and impurities that interfere with GC–MS analysis. For quantitative analysis, β-caryophyllene standard and essential oil were dissolved with ethyl acetate and analyzed using a GC-MS-QP2010 (Shimadzu, Japan) equipped with an AOC-20i autosampler. One microliter of the essential oil was injected, and the chromatographic separation of β-caryophyllene was achieved with HP5-MS column (60 m × 0.25 mm I.D. × 0.25 mm thickness; Restek, United States). Helium with a purity of 99.999% was utilized as the carrier gas at a flow rate of 10 ml/min.

The injector port, interface, and ion source temperatures were set at 260, 300, and 230°C, respectively. The programmed oven temperature was as follows: the initial temperature was set at 120°C for 1 min, then raised to 270°C at 6°C/min and finally to 320°C at 7°C/min, and held for 10 min. A solvent delay of 6 min was set to protect the filament from oxidation. The mass spectrometer was operated in electron impact (EI) mode at an ionization potential of 70 eV and at an emission current up to 60 mA. The quantification was performed in selected ion monitoring (SIM) mode by monitoring mass ions for each β-caryophyllene. For quantification, four different calibrators (0.125, 0.250, 0.500, and 1.00 mg/ml) were used for linearity check. It showed good correlation coefficient (*r*^2^ = 0.9999). Scanning interval and SIM sampling rate were 0.5 and 0.2 s, respectively.

### Statistical Analysis

The evaluation of β-caryophyllene from various callus and HR samples of *A. vulgaris* L. leaf explants was performed with three replicates each. The data obtained were analyzed statistically, and observations were recorded on the frequency of both FW and DW (callus and HR cultures, respectively), as well as the number of explants responding for HR initiation growth rate. The statistical analyses were performed by ANOVA using SPSS. The differences between means were tested for significance by Duncan’s multiple range test at *p* = 0.05.

## Results and Discussion

### Callus Induction

**FIGURE 2:**
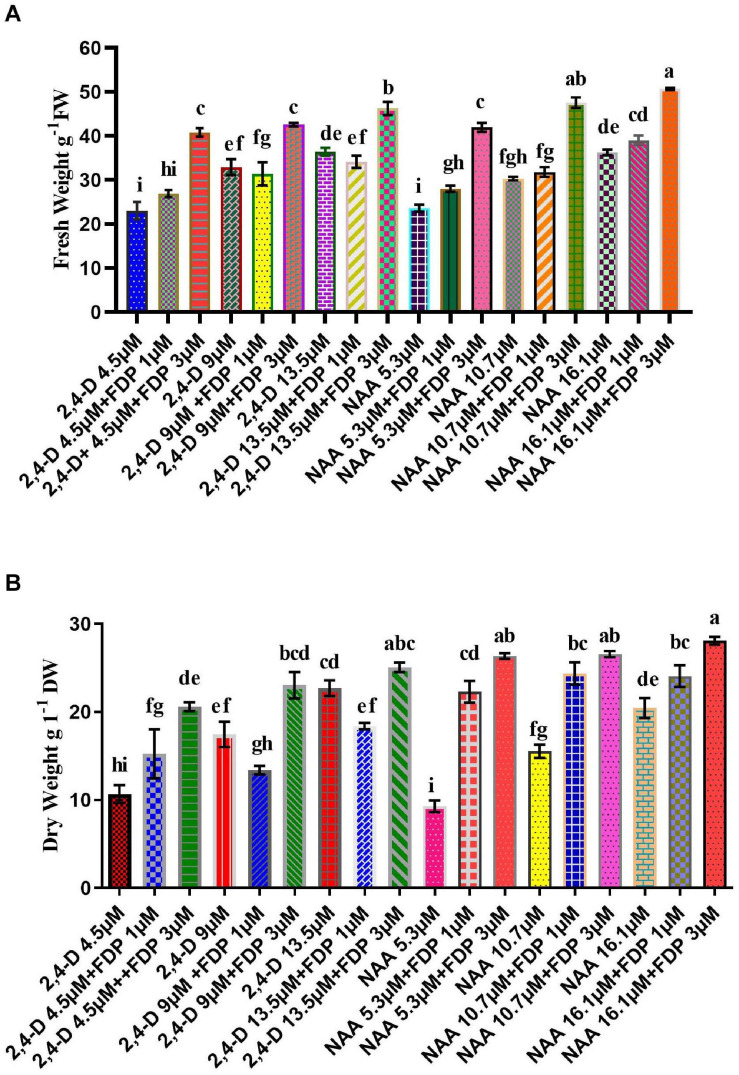
Productivity of precursor treatment of callus culture yield and biomass cultivation. **(A)** Various concentrations of 2,4D + FDP and NAA + FDP treatments of fresh weight of callus. **(B)** Various concentrations of 2,4D + FDP and NAA + FDP treatments of dry weight of callus.

Leaf explants were inoculated into MS medium containing 2,4D (4–13 μM) and NAA (5–16 μM) with FDP as a precursor (1 and 3 μM) individually, as per a previous study by Sundararajan and Kumari (2015). Among various concentrations tested, the highest response of callus (99 and 95.2%) was achieved in MS medium supplemented with a combination of 2,4D (9.0 μM) + FDP (3 μm), followed by MS medium supplemented with NAA (16.1 μM) + FDP (3 μM). There are various similar studies on callus induction from leaf and stem explants of *A. vulgaris* L. (Sujatha and Kumari, 2007; Borzabad et al., 2010a, b). The precursor- and elicitor-treated calli vary from species to species and often differ even within the same species, for example, *Jatropha integerrima* (Schmitt and Petersen, 2002). MS medium supplemented with 5-aminolevulinic acid (5-ALA) as a precursor 2, 5, and 10 mg/L concentration has shown an enhancement in the callus biomass and induction of rooting and shooting with a profound effect (Boitel-Conti et al., 2000). Sujatha and Dhingra (1993) reported that feeding the yeast extract elicitor in the suspension culture of *J. integerrima* promoted the metabolic enzyme activity and alkaloid production several folds. Other studies also reported enhanced biosynthesis of the flavonolignan silymarin compound through jasmonic acid (JA) elicitor treatment of *Silybum marianum* callus cell culture medium (Sanchez-Sampedro et al., 2005). In recent reports, three key genes, such as phenylalanine ammonia-lyase, chalcone synthase, and dihydroflavonol-4-reductase, were reported to be involved in the phenylpropanoid pathway. A polyphenolic compound has been used in *Plantago ovata* callus culture media with different growth regulators (Talukder et al., 2016). Arya and Patni (2013) reported significant enhancement of quercetin in callus culture of *Pluchea lanceolata* by incorporation of cinnamic acid and precursor feeding with L-phenylalanine. In the present study, FW/DW of callus 2,4D treated without precursor was observed at FW/DW: 36/22 g, whereas in precursor-treated callus the highest FW/DW: 48/28 g were noticed at the concentrations (2,4D 13.5 μM + FDP 3 μM) followed by (2,4D 9 μM + FDP μM) fresh and dry weight was exhibited (Figure 2A). Then fresh and dry weight of NAA treated callus without precursor was found to be 36/20 g, respectively, whereas in precursor-treated callus the highest FW/DW: 48/26 g was noticed at the concentrations (NAA 16.1 μM + FDP 3 μM) followed by (NAA 10.7 μM + FDP 3 μM) fresh and dry weight was exhibited (Figure 2B). Similar reports were observed in connection with FW 500 mg withanolides production in callus culture of *Withania somnifera* after 4 weeks of culture (Doma et al., 2012; Sivanandhan et al., 2012). This study is in agreement with the result of a previous report on the large-scale production of artemisinin with different elicitors and precursors, such as sodium acetate, mevalonic acid, lactone, and casein acid hydrolysate, to increase artemisinin content in cell suspension culture of *Artemisia annua* (Baldi and Dixit, 2008). Quercetin compound was enhanced by using various precursors, such as phenylalanine and cinnamic acid, in *P. lanceolata in vitro* callus culture (Arya and Patni, 2013). Similar results were also observed in *Psoralea corylifolia* with precursor treatment in cotyledonary callus, which was obtained from FW 2,601.8 mg/g (Mohammadparast et al., 2015). A similar phenomenon was also observed with different synthetic precursors, such as phenobarbital, geraniol, *trans*-cinnamic acid, succinic acid, and tryptamine, in compact callus cluster (CCC) cultures of *Catharanthus roseus* with the highest biomass production of FW 10 g and DW 6 g (Zhao et al., 2001). Phenylethanoid glycosides were enhanced by feeding precursor into the *Cistanche deserticola* cell culture medium, with FW 0.6 g and DW 0.4 g (Ouyang et al., 2005). In earlier studies, the intracellular amount of oleanolic acid (OA) reached its final value (DW 0.84 mg/g) after 72 h of treatment with 100 M JA, which was 9.4-fold greater than that observed in untreated control cultures. After 48 h of therapy, the inclusion of chitosan at 50 mg/L induced a 5-fold increase in OA accumulation (DW 0.37 mg/g) (Wiktorowska et al., 2010). The precursor feeding or elicitor treatment is an effective technique to improve the growth of secondary metabolites through plant cell culture (Dedaldechamp and Uhel, 1999 and Rao and Ravishankar, 2002).

### HR Culture

#### Induction and Establishment of HR Culture

**FIGURE 3:**
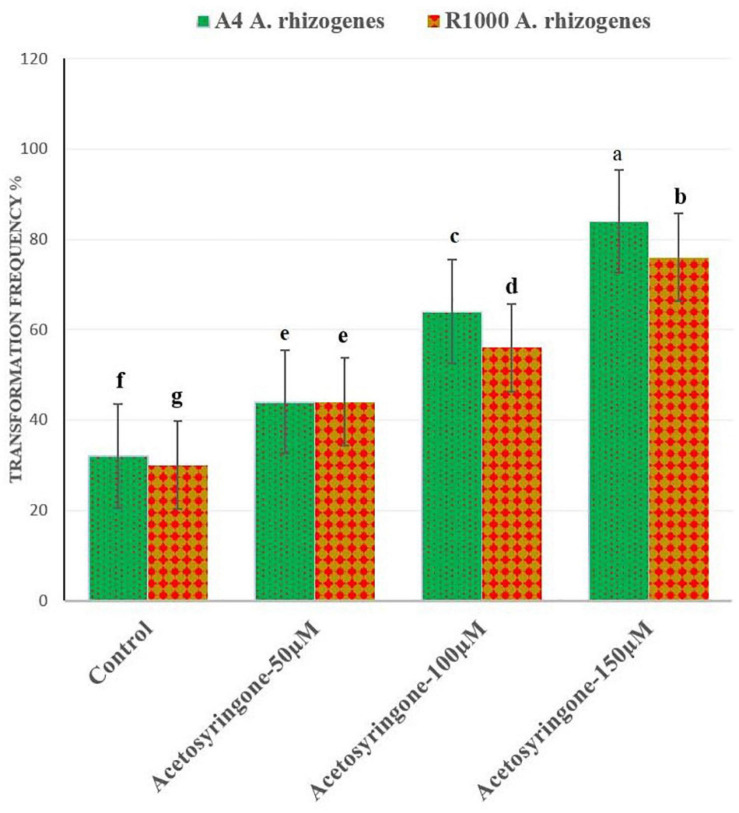
Effect of different concentrations of acetosyringone (AS) on *A. rhizogenes* (A_4_ and R_1000_ strains) transformed frequency (%) of hairy roots inductions in *A. vulgaris*.

The transformation efficiency of *A. rhizogenes* required a different time and duration for the root induction from leaf explants of *A. vulgaris* L. (Table 1). The highest transformation frequency with a direct root induction from the wounding site was observed in leaf explants infected with AS 150 μM (A_4_ and R_1000_) strains after 10–15 days of culture (Figure 3). The results of the present study were similar to *Persicaria minor* HR induction treatments with AS at 100 and 200 μM, which suggested that phenolic compounds play a vital role in the induction medium, particularly at wounded sites (Danphitsanuparn et al., 2012; Ashraf et al., 2015). It was reported that AS stimulated *A. rhizogenes* T-DNA to bring out HR from the wounded cell tissue (Farshad Ashraf et al., 2013). HR induction by different bacterial strains has been documented earlier in different plant species (Zehra et al., 1999). In *C. roseus*, six different *A. rhizogenes* strains were used for HR induction (Toivonen et al., 1989). Strain induced HR with the help of *Rauwolfia serpentina* leaf explants with a transformation frequency of 70% for strain when compared with strain LBA 9402, with a frequency of 45% (Goel et al., 2010). Tao and Li (2006) reported HR induction in *Torenia fournieri* leaf explant with higher transformation efficiency at 65% than that in A_4_ strain. Wu et al. (2003) earlier reported that 100 μM of AS is the best concentration for enhancing the genetic transformation in *Nicotiana tabacum* var. Previous studies indicated that the *A. rhizogenes* strain, which influenced HR induction in various explant parts, was showing HR induction (Wu et al., 2003; Pietrosiuk et al., 2006; Thimmaraju et al., 2008). In the present study, exogenous application of AS along with naturally produced phenolics from wounded leaf explants increased the transformation frequency by vir-genes induction.

**TABLE 1:** Frequency of hairy root induction on leaf explants derived from *A. rhizogenes* strains.

Bacterial strain	Explants type	Frequency of transformation%	Hairy root induction (days)
A_4_	Leaf explants	93.0 ± 0.4	11
R1000	Leaf explants	85.9 ± .0.2	

*Data are means of three replicates and ± is SE.*

#### Analysis of the GI of HRs

**FIGURE 4:**
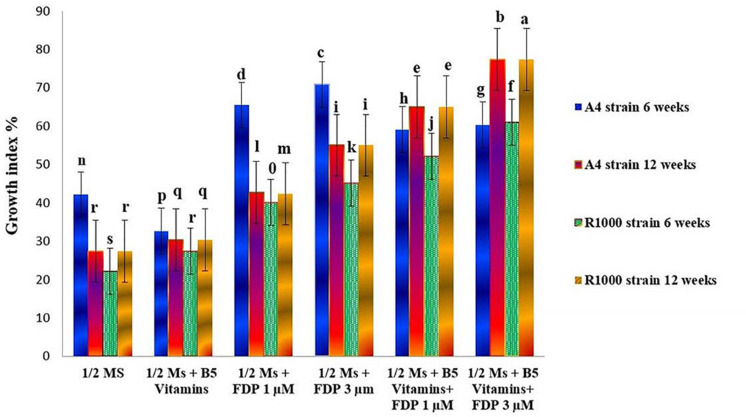
Effect on the mean growth index of *A. vulgaris* hairy root culture after 6 and 12 weeks (A_4_ and R_1000_ strains).

**FIGURE 5:**
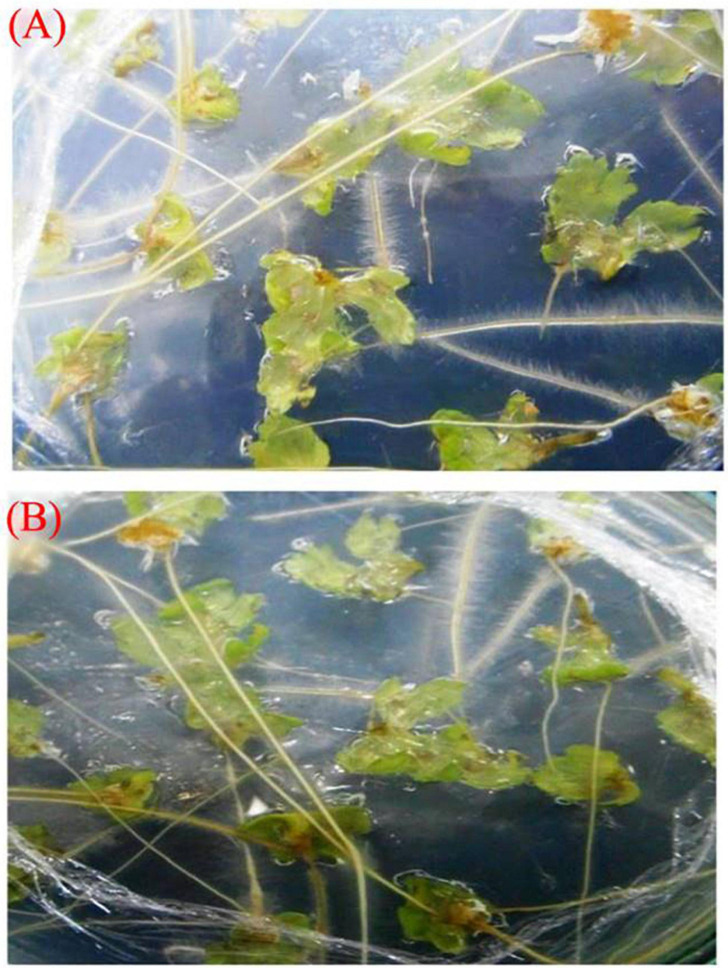
**(A)** After 2 weeks of coculture of A_4_ strain on hairy root initiation from leaf explants cultured on MS + 500 mg/L cefotaxime medium. **(B)** After 2 weeks of coculture of R_1000_ strain on hairy root initiation from leaf explants cultured on MS + 500 mg/L cefotaxime medium.

**FIGURE 6:**
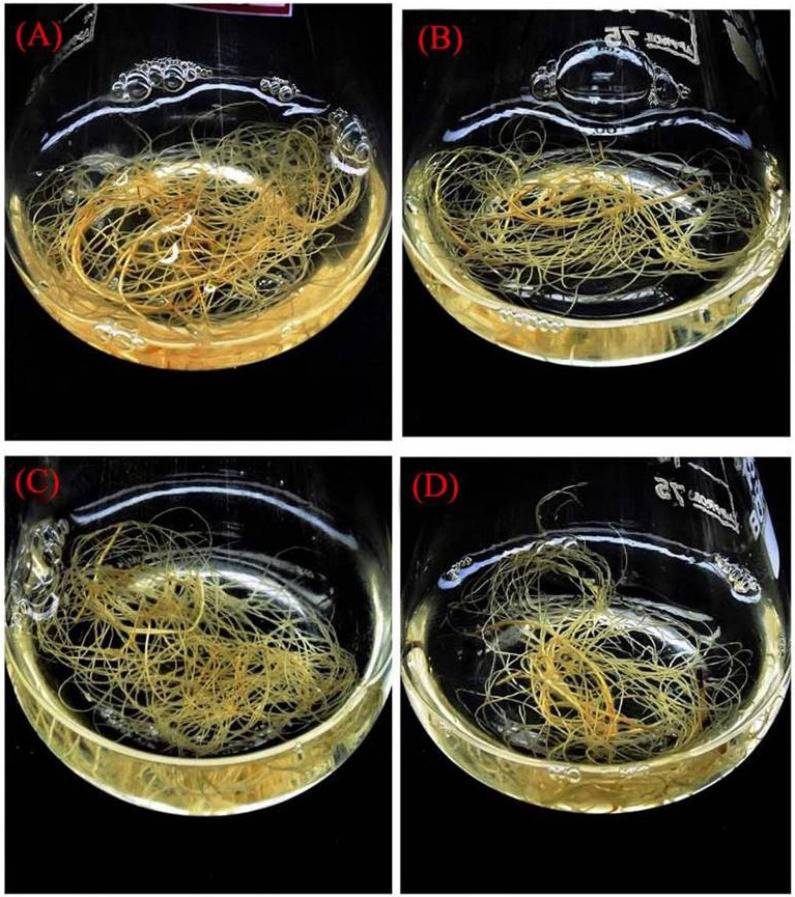
Enhancement of precursor treated with biomass accumulation from hairy root culture after 12 weeks. **(A)** A_4_ strain hairy root with 1/2 MS + FDP 3 μM. **(B)** A_4_ strain hairy root with 1/2 MS + FDP 1 μM. **(C)** R_1000_ strain hairy root with 1/2 MS + FDP 3 μM. **(D)** R_1000_ strain hairy root with 1/2 MS + FDP 1 μM.

**FIGURE 7:**
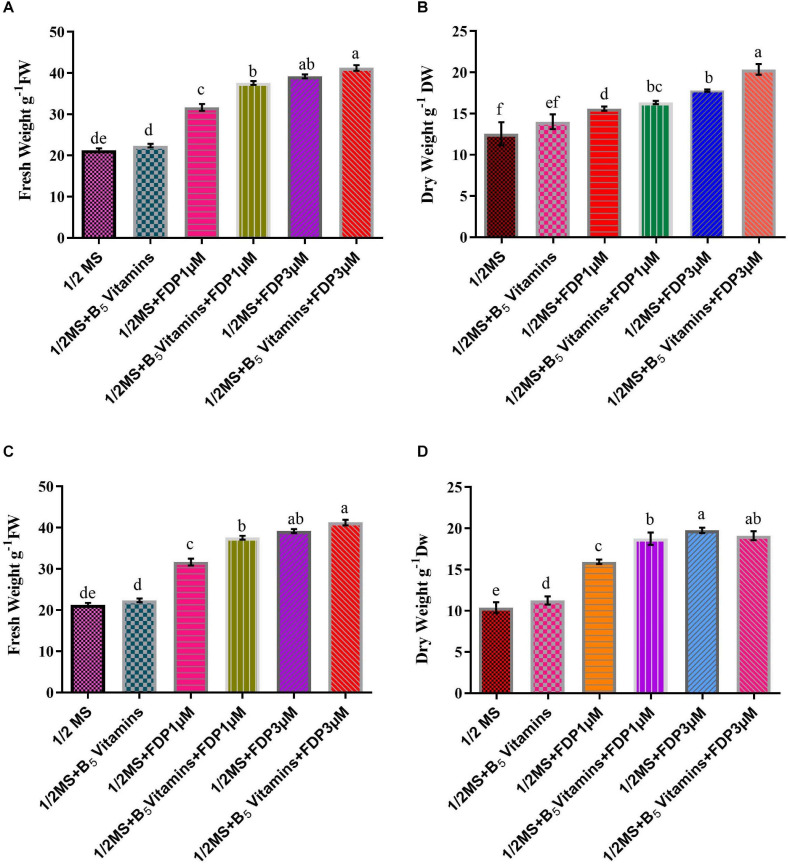
Productivity of precursor treatment of hairy root culture yield and biomass cultivation. **(A)** Various concentrations of A_4_ strain treatment of fresh weight of hairy root culture. **(B)** Various concentrations of A_4_ strain treatment of dry weight of hairy root culture. **(C)** Various concentrations of R_1000_ strain treatment of fresh weight of hairy root culture. **(D)** Various concentrations of R_1000_ strain treatment of dry weight of hairy root culture.

There are various compositions and types of culture medium known to influence the growth and production of HRs (Giri and Narasu, 2000). Among all the treatments tested (1/2 MS, 1/2 MS + B_5_ vitamins, 1/2 MS + FDP 1 μM, 1/2 MS +FDP 3 μM, 1/2 MS + B_5_ vitamins + FDP 1 μM, and 1/2 MS + B_5_ vitamins + FDP 3 μM), the HR culture attained a maximum and minimum of a 30-fold and a 65-fold increase of the initial weight of 200 mg (strain), and the HR culture attained a maximum and minimum of a 25-fold and a 55-fold increase in the initial weight of 163 mg (R_1000_) within 12 weeks on 1/2 MS + FDP 1 μM and 1/2 MS + FDP 3 μM, respectively. After 6 weeks, there was no significant difference in the GI% in another precursor-treated HRs. However, after 12 weeks of culture, GI% was greater in the GI of *A. vulgaris* L. HRs with a strain at 6 and 12 weeks in the rotary shaking system. At 6 weeks GI of A_4_ HR culture, GI% was found without precursor treatment in 1/2 MS (42.10%) and 1/2 MS + B_5_ vitamins (32.55%) and with precursor treatment in 1/2 MS + FDP 3 μM (70.85%) and 1/2 MS + B_5_ vitamins + FDP 3 μM (60.23%). After 12 weeks, the GI of A_4_ HR culture without precursor treatment was observed as 1/2 MS (27.33%) and 1/2 MS + B_5_ vitamins (30.33%), and with precursor treatment, it was observed with the highest response in 1/2 MS + FDP 3 μM (55.00%) and 1/2 MS + B_5_ vitamins + FDP 3 μM (77.33%), respectively (Figures 4, 5A, 6A,B). Figure 4 shows the GI of *A. vulgaris* L. by HRs at 6 and 12 weeks cultivation in the rotary shaking system. At 6 weeks, the GI of R1000 HR culture without precursor treatment was observed in 1/2 MS (22.11%) and 1/2 MS + B5 vitamins (27.44%), and with precursor treatment, it was observed in 1/2 MS + FDP 3 μM (45.11%) and 1/2 MS + B5 vitamins + FDP 3 μM (61.00%). After 12 weeks, the GI of R1000 HR culture without precursor treatment was observed in 1/2 MS (27.33%) and 1/2 MS + B5 vitamins (30.31%), and with precursor treatment, it was observed in 1/2 MS + FDP 3 μM (55.00%) and 1/2 MS + B5 vitamins + FDP 3 μM (77.33%) (Figures 5B, 6C,D). In an earlier report, *A. vulgaris* L. HR culture grown in different liquid culture media, such as B5, SH (Schenk and Hilderbrandt), MS, and 1/2 MS, after 30 days showed 3-fold increases in growth rate (Sujatha et al., 2013). Similarly, HRs grown in full-strength and half-strength MS media, respectively, showed 46- and 34-fold increased growth rates in *C. roseus* L. plant (Hanafy et al., 2016). In another study, *A. rhizogenes* MTCC 532 used in HR induction with the combination of 1/2 MS liquid medium without hormones showed GI with a 45-fold increase within 57 days of culture (Khatodia et al., 2013). In *Gentiana scabra* HR culture, a maximum 42-fold increase was achieved through B5 liquid media composition after 8 weeks of cultivation (Huang et al., 2014). The establishment of HR liquid cultures was observed in *Rauvolfia micrantha* (Sudha et al., 2003) and *Gentiana macrophylla* (Tiwari et al., 2007). Inconsistent with the above work, the present study noticed a higher growth rate index of HRs in *A. vulgaris* L. on the same B5 and 1/2 MS liquid medium compositions. The HRs grown in 1/2 MS + B5 vitamins + FDP 3 μM medium showed a higher biomass increase and were expressed in either FW or DW when compared with those grown in 1/2 MS, 1/2 MS + B5 vitamins, 1/2 MS +FDP 1 μM, 1/2 MS + B5 vitamins + FDP 1 μM, and 1/2 MS + FDP 3 μM. A4 strain HR production increased with FW and DW on different media, such as FW 1/2 MS (23 g), 1/2 MS + B5 vitamins (26 g), 1/2 MS + FDP 3 μM (39 g), and 1/2 MS + B5 vitamins + FDP 3 μM (40 g) and DW 1/2 MS (12 g), 1/2 MS + B5 vitamins (14 g), 1/2 MS + FDP 3 μM (17 g), and 1/2 MS + B5 vitamins + FDP 3 μM (20 g). The R1000 HRs showed high production with increased FW and DW on different media: FW 1/2 MS (21 g), 1/2 MS + B5 vitamins (22 g), 1/2 MS + FDP 3 μM (39 g), and 1/2 MS + B5 vitamins + FDP 3 μM (41 g) and DW 1/2 MS (10 g), 1/2 MS + B5 vitamins (11 g), 1/2 MS + FDP 3 μM (19 g), and 1/2 MS + B5 vitamins + FDP 3 μM (19 g) (Figure 7). The present findings are in agreement with the report of Huang et al. (2014) who stated that the use of HR cultures is an excellent alternative to harvesting natural *in vitro* grown plants to produce pharmaceuticals and important metabolites in less time with the highest quality. In *Salvia officinalis* L. HR culture with strain, the maximum accumulated DW was achieved with 45 mg for 50 days (Grzegorczyk et al., 2006). The largest biomass was enhanced with 1/2 MS medium of low sucrose concentration to increase the lateral branches. In agreement with the findings, sucrose has been reported to increase HR biomass in *Artemisia absinthium* (Nin et al., 1997) and *Swertia chirata* (Keil et al., 2000). Inoguchi et al. (2003) also reported that high-level growth rates of root were induced by using 1/2 MS salt liquid medium in *Solidago altissima*. In *Callerya speciosa*, the highest growth of HRs was in 1/2 MS medium 10.85 g of FW and 1.13 g of DW and B5 medium, followed by 7.50 g of FW and 0.63 g of DW (Yao et al., 2016). *Perilla frutescens* cells of 7-day-old cultures were exposed to a yeast elicitor at 0.5–5% *(v/v)* in 7 days. Anthocyanin content peaked around 10.2% DW with yeast elicitor at 1% *(v/v)*. Simultaneously, the maximum production of OA and ursolic acid was 19 and 27 mg/L, respectively, and a 46 and 24% increase in cultures administered with a 2% *(v/v)* yeast elicitor (Wang et al., 2004).

### PCR Analysis

**FIGURE 8:**
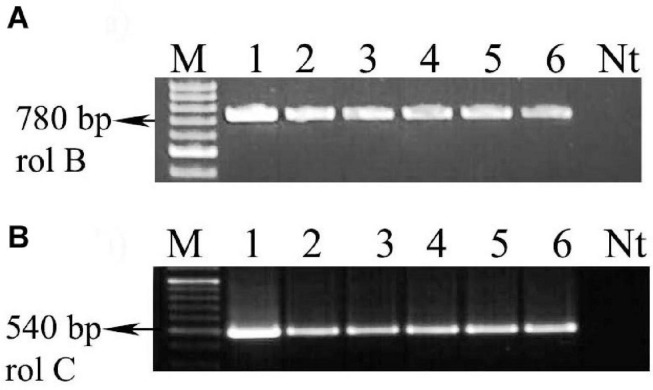
PCR conformations of transgenic hairy roots (A_4_ and R_1000_) from *A. vulgaris* L. leaf explants. **(A)** *rolB* gene amplification (780 bp). **(B)** *rolC* gene amplification (540 bp). Lane M: marker, lane 1: A_4_ plasmid DNA, lane 2: R_1000_ plasmid DNA, lanes 3 and 4: genomic DNA of hairy root culture (A_4_ strain), lanes 5 and 6: genomic DNA of hairy root culture (R_1000_ strain), Nt: non-transgenic root.

Molecular confirmation of the transformed HRs was achieved by PCR analysis. The presence of *rolB* gene fragment (780 bp) and *rolC* gene fragment (540 bp) was confirmed by using gene-specific primers, and successful transformation was confirmed by the presence of *rolB* and *rolC* gene fragments. The results of PCR study indicated that the *rolB* and *rolC* gene fragments were incorporated into the *A. vulgaris* L. HR cultures. It indicates that the presence of the incorporation of the T-DNA into the genome of *A. vulgaris* L. HRs has been successful (Figure 8 and Supplementary Figure 5). Many studies concluded that the HR culture metabolite production depends on *rol* gene expression (Tiwari et al., 2007; Bulgakov, 2008). The *rol* genes play a major role in the pathway that leads to the increased production of secondary metabolites. In many cases, the *rol* gene of strain is involved in the parthenogenesis of the HR disease and establishment of transgenic plants with simple developmental and morphological alteration (Jouanin et al., 1987). The establishment of the root inducing (Ri) disease in HRs is interrelated with the expression of the *rolA*, *B*, *C*, and *D* loci in the majority assay of plant species (Taylor et al., 1985; Cardarelli et al., 1987). Along with them, *rolB* plays the most important role, whereas *rolA*, *rolC*, and *rolD* encourage the root development synergistically (Aoki and Syôno, 1999). The growth model of *rolB*-induced HR is characterized by high-level growth, branching, and plagiotropism (Capone et al., 1989). Genetically mediated transformation by Ri T-DNA of *A. rhizogenes* has been established to be an effective indirect way of accumulating and producing high levels of secondary metabolites in plant cells (Guillon et al., 2006). In a confirmation study in agreement with *A. rhizogenes*, there is a type of Gram-negative well-known soil-borne bacterium that causes HRs in many plants. Root loci (*rol*) genes harbored by the Ri plasmid of this bacterium are integrated into the host plant genome, causing HRs. *rol* genes are a reflection to affect the growth and development of transformed roots and induce secondary metabolite synthesis by turning on the mediated transcription defense genes (Ono and Tian, 2011).

### Isolation of Essential Oil and Quantification of β-Caryophyllene Content

#### Quantification of β-Caryophyllene in Callus Culture

The essential oil isolated from *A. vulgaris* L. callus was quantified with various concentrations of the β-caryophyllene standard compound. The calibration curve (SIM method) was determined to be utilized. GC–MS of β-caryophyllene compound retention time peak was observed at 7.34 s. In the present study, extracts of callus samples were analyzed through GC–MS by the scan mode method, and the highest β-caryophyllene compound was observed in without precursor-treated callus 2,4D 13.5 μM (1.61 mg/ml) and precursor-treated callus 2,4D 13.5 μM + FDP 3 μM (2.38 mg/ml), followed by NAA 16.1 μM (2.28 mg/ml) (Supplementary Figures 1, 2 and Table 2). These results were supported by a previous report (Arya and Patni, 2013), where enhanced quercetin compound (0.23 mg/ml) in precursor-treated callus was noticed. Treatment of *A. absinthium* callus with benzyl adenine (BAP) and NAA (2 mg/L), along with amino acids, such as valine and cysteine (12.5 mg/L), resulted in higher content of artemisinin compound (Zia et al., 2007). Enhanced trigonelline content was noticed through thin-layer chromatography, gas–liquid chromatography, and GC–MS analysis in nicotinic acid used as precursor in *in vitro* callus culture (Mathur and Kamal, 2012). Similarly, casein hydrolysate precursor feeding in cell suspension culture at 50, 75, and 100 mg/L concentrations resulted in the highest scopoletin compound, as revealed by high-performance thin-layer chromatography analysis (Abyari et al., 2016). Various explants of *Simmondsia chinensis* (leaves, nodes, internodes, shoot apices, and cotyledons) were used for callus induction from MS media along with various growth regulators [NAA, BAP, indole-3-acetic acid (IAA), and isopentenyl adenine (2ip)], and the final quantitative estimation of essential oils was observed at the highest amount of oil content at a 9-week-old callus culture (Aftab et al., 2008). A similar phenomenon has been observed in several studies (Zhao et al., 2001; Ouyang et al., 2005; Sivanandhan et al., 2012). In this study, calli are compared with previous reports that were treated with different precursors/elicitors as shown in Table 3.

**TABLE 2:** Comparative study on various precursor/Elicitor treatment of different types of plant species.

Species	TreatmentPrecursor/Elicitor	Tissue/culture	Metabolites	Concentrations	Analysis method	References
*Artemisia annua*	Mevalonic acid lactone	Callus culture	artemisinin	50 mg/mL	HPLC	Baldi and Dixit, 2008
*Panax notoginseng*	2-hydroxyethyl jasmonate (HEJA)	Callus culture	ginsenoside	3.6 mg/mL	HPLC	Wang and Zhong, 2002
*Saussurea medusa*	cinnamic acid	Callus culture	flavonoids	1.8 mg/mL	HPLC	Lu et al., 2001
*Hypericum perforatum*	L-phenylalanine	Organogeneis culture	Hyperforin	150 mg/mL	HPLC	Liu et al., 2007
*Psoralea corylifolia*	Umbelliferone	Callus culture	psoralen	25 mg/mL	TLC	Parast et al., 2011
*Plumbago rosea*	Jasmonic acid	Adventitious root cultures	Plumbagin	1.23%	HPLC	Silja and Satheeshkumar, 2015
*Zingiber zerumbet*	Carbohydrate substrate	Callus culture	Zerumbone	3.90 mg/L	HPLC	Jalil et al., 2015
*Ammi majus*	Enterobacter sakazaki	Hairy root culture	umbelliferone	2.2 mg %	GC-MS	Staniszewska et al., 2003
*Pluchea lanceolata*	Cinnamic acid	Callus culture	phenylalanine	5 mg/mL	HPLC	Arya et al., 2008
*Artemisia annua*	Farnesyl pyrophosphate	Hairy root culture	artemisinin	10.3 mg/L	HPLC	Ahlawat et al., 2014
*Vinca minor*	Naproxen+hydrogen peroxide+aceticanhydride+ tryptophan+secologanin	Hairy root culture	vincamine	0.017 %	HPLC	Verma et al., 2014
*Moringa oleifera*	Nicotinic acid	Callus culture	Trigonelline	2.73 mg/g	GC-MS	Mathur and Kamal, 2012
*Valeriana officinalis*	Magnesium and calcium abiotic elicitors	Hairy root culture	valerenic acid	1.83 mg/g	HPLC	Torkamani et al., 2014
*Withania somnifera*	ASA	Hairy root culture	withaferin A	171 μg/g	HPLC	Doma et., al 2012
*Spilanthes acmella*	L-phenylalanine	Callus culture	scopoletin	27.12 mg/g	HPTLC	Abyari et al., 2016
*Rauvolfia tetraphylla*	2,4-D+ tryptophan	Callus culture	Reserpine	2.1 mg/g	HPLC	Anitha and Ranjitha Kumari, 2006

**TABLE 3:** Different concentration of with and without precursor treated callus samples quantified by GC-MS.

Various precursor treatment of calli (μM/mL)	β-caryophyllene quantitative concentration (mg/mL)
**2,4-D**	
4.5	0.45
9.0	0.93
13.5	1.61

**2,4-D +,4-D**	
4.5+.	0.65
9.0+.	0.95
13.5+3	1.98
4.5+.	1.99
9.0+.	2.10
13.5+3	2.38

**NAA**	
5.3	1.35
10.7	1.47
16.1	2.28

**NAA+AA8**	
5.3+.	1.52
10.7+0	1.78
16.1+6	1.98
5.3+.	1.62
10.7+0	2.02
16.1+6	2.25

#### Quantification of β-Caryophyllene in HR Culture

The essential oil isolated from the HR was quantified with various concentrations of the β-caryophyllene standard compound by the SIM method. The retention time of β-caryophyllene compound was observed at 7.34 s. Among various treatments of the A_4_ induced HR, the highest β-caryophyllene compound in HR cultures without precursor treatment was 2.44 mg/ml, whereas precursor-treated HR showed 2.92 mg/ml (Supplementary Figure 3 and Table 4). The highest β-caryophyllene compound in R_1000_ induced HR cultures without precursor treatment was 1/2 MS (2.16 mg/ml) and precursor treatment with 1/2 MS + FDP 3 μM (2.80 mg/ml) (Supplementary Figure 4 and Table 4). The observation was in accordance with the results of *P. minor* HR induction from different *A. rhizogenes* strains (A_4_, ATCC43056, ATCC15834, and ATCC13333) (Ashraf et al., 2015). FDP precursor could be suitable for *in vitro* secondary metabolites engineering strategies for increasing β-caryophyllene content (17%) that was found in *P. minor* roots (Ismail et al., 2011), *Micromeria fruticosa* (Lu et al., 2001), *Copaifera* species (Liu et al., 2007), *Litsea cubeba* (Parast et al., 2011), *Ailanthus altissima* (Ayeb-Zakhama et al., 2014), *Rosmarinus officinalis* L. (Flamini et al., 2015), and *Polygonum minus* (Baharum et al., 2010). Recently, β-caryophyllene synthase gene (*QHS1*) from *A. annua* was confirmed in the genome of the *Cyanobacterium synechocystis* species by means of gas chromatography with flame-ionization detection (GC-FID) and GC–MS analyses (Cai et al., 1994; Reinsvold et al., 2011).

**TABLE 4:** Different concentrations of with and without precursor treated hairy root samples quantified by GC-MS.

Various precursor treatment of hairy root culture (μM/mL)	β -caryophyllene quantitative concentration (mg/mL)
**A_4_ strain**	
½ MS	2.44
½ MS+B_5_ Vitamins	2.12
½ MS+FDP1	2.62
½ MS+FDP 3	2.92
½ MS+B_5_Vitamins + FDP1	2.31
½ MS+B_5_ Vitamins+ FDP 3	2.57

**R_1000_ strain**	
½ MS	2.16
½ MS+B_5_ Vitamins	2.05
½ MS+FDP1	2.38
½ MS+FDP 3	2.80
½ MS+B_5_Vitamins + FDP1	2.10
½ MS+B_5_ Vitamins+ FDP 3	2.25

Similarly, a farnesyl pyrophosphate synthase (VoFPS) gene co-transformation system was developed in *Valeriana officinalis* HRs for the enhancement of β-caryophyllene compound by recombinant and elicited methods (Ricigliano et al., 2016). The attained values, similar to those reported from different *A. rhizogenes* mediated transformations of several medicinal plant species, have been studied for the production of essential oil by both transformed and non-transformed root cultures (Santos et al., 2002, 2005; Szoke et al., 2004; Sujatha et al., 2013). The results from the present study agreed with the previous reports on various precursor and elicitor treatments for root induction. Some plants of vincamine biosynthesis were established through *Vinca minor* HR clone that was determined with various elicitor (acetyltransferase) and precursor treatments (cyclooxygenase inhibitor); the highest vincamine compound was observed at 17% (Verma et al., 2014). Enhanced tanshinones production in *Salvia miltiorrhiza* HR culture was used with different elicitors combined with yeast extract **+** Ag**^+^** and Ag**^+^ +** methyl jasmonate (MeJA) for yeast extract **+** Ag**^+^ +** MeJA, and elicitation metabolite profiling was analyzed by ultra-performance liquid chromatography (UPLC) (Cheng et al., 2013). The use of MeJA and other signaling compounds for elicitation of transforming root cultures is opening a new way for a possibly profitable *in vitro* secondary metabolite production (Ahlawat et al., 2014). A soluble recombinant enzyme can catalyze the covalently bonded metal ion-dependent conversion of FDP to β-caryophyllene, sesquiterpene olefin contained in *A. annua* essential oil, was given by heterologous gene expression in *Escherichia coli.* Moreover, all through the initial stages, the β-caryophyllene synthase overexpressed in certain plant tissues and triggered in old ones in response to fungal elicitor suggests a role besides β-caryophyllene in host defense (Cai et al., 2002). Recently, Souret et al. (2002) have cloned three genes from *A. annua* that affect terpenes’ biosynthesis: 1-deoxy-D-xylulose-5-phosphate synthase (DXS), 1-deoxy-D-xylulose-5-phosphate reductoisomerase (DXR), and synthase from squalene (SQS) to use a library of cDNA made from hairy *A. annua* roots. Previously, overexpression of farnesyl pyrophosphate synthase in *A. annua* leads to high artemisinin aggregation levels by converting IPP and DMADP into diphenylphosphinic acid pentafluorophenyl ester (FDPP) (Banyai et al., 2010). Cloning and characterization of *A. annua* 1-hydroxy-2-methyl-2-(E)-butenyl 4-diphosphate reductase (AaHDR) involved in enhanced artemisinin production *via* improved artemisinin biosynthetic pathway precursor development through the MEP pathway were being reported in *A. annua*. Tryptophan alkaloid compound enhanced through precursor feeding in HR culture has been identified (Hughes et al., 2004). The effect of precursor-treated HR suspension culture has produced a high level of glucosinolate compound of *Nasturtium montanum* and *Cleome chelidonii* (Songsak and Lockwood, 2004). Robins et al. (1990, 1991) also reported hyoscyamine compound biosynthesis during metabolic pathways as amino acid precursors. *A. rhizogenes* enables the high production of these secondary metabolites for extensive industrial applications (Srivastava and Srivastava, 2007; Suza et al., 2008; Swarna and Ravindhran, 2012) that are useful as pharmaceuticals, cosmetics, and food additives (Signs and Flores, 1990; Guillon et al., 2006). In this study, HR culture has been compared with some previous reports that were treated with different precursors/elicitors (Table 3).

## Conclusion and Perspectives

This study, to the best of our knowledge, is the first attempt in reporting *in vitro* precursor treatment for enhancement of β-caryophyllene content by using callus and HR culture of *A. vulgaris* L. Although the biosynthesis pathway of secondary metabolites has not been well explored, the terpenoid biosynthesis pathway is well known for the production of β-caryophyllene *via* FDP. In this study, we aimed to enhance the output by using FDP as a precursor, and our findings proved that by using precursor-treated cultures, we can improve β-caryophyllene production. In conclusion, β-caryophyllene enhancement was successfully done with precursor-treated HR culture of *A. vulgaris* L. after 12 weeks in combined treatment culture of 1/2 MS + FDP 3 μM (2.92 mg/ml, respectively). These findings could encourage and provide support to synthesize new active natural bioactive compounds as an alternative to synthetic repellent and insecticides.

## Data Availability Statement

The original contributions presented in the study are included in the article/Supplementary Material, further inquiries can be directed to the corresponding author/s.

## Author Contributions

SB and BR conceived the idea presented. SB developed the study, performed the experimental work, and wrote the main manuscript. RB developed the GC–MS methodology and performed the analysis. AM and DS analyzed the data interpretation. SS and GP revised the manuscript contents. Finally, all authors have read and approved the manuscript.

## Conflict of Interest

The authors declare that the research was conducted in the absence of any commercial or financial relationships that could be construed as a potential conflict of interest.
