# Supplementary material for: Enhanced Production of β-Caryophyllene by Farnesyl Diphosphate Precursor-Treated Callus and Hairy Root Cultures of Artemisia vulgaris L
Source: Front Plant Sci. 2021 Mar 30;12:634178. doi: 10.3389/fpls.2021.634178 (PMC8042329; doi:10.3389/fpls.2021.634178)
Supplement: Supplementary file 1 [file Data_Sheet_1.doc]

**Enhanced production of β-Caryophyllene by Farnesyl Diphosphate precursor treated callus and hairy root cultures of *Artemisia vulgaris* L.**

**B. Sundararajan^†*^, B. D. Ranjitha Kumari^‡^, Anilkumar Moola^‡^, D.Sathish**^¶^**, G.Prem Kumar^§^, S. Srimurali^ǁ^, and R. Babu Rajendran┴**

**^†^***College of Horticulture and Landscape Architecture, Southwest University,*

*Chongqing-400716, P.R.China*

**^‡^***Department of Botany, Bharathidasan University, Tiruchirappalli-620 024, Tamil Nadu,*

*India*

^¶^*Department of Biotechnology, Bharathidasan University, Tiruchirappalli-620 024, Tamil*

*Nadu, India*

**^§^***China-USA Citrus Huanglongbing Joint Laboratory,National Navel Orange Engineering*

*Research Center, Gannan Normal University,Ganzhou City, Jiangxi Province,*

*P.R.China-341000*

**^ǁ^***ICMR-NIN Hyderabad- 500007, Telangana, India*

**┴***Department of Environmental Biotechnology, Bharathidasan University,*

*Tiruchirappalli-620 024, Tamil Nadu, India.*

*Correspondence to- sundarpbt87@gmail.com; ranjithakumari2004@yahoo.co.in


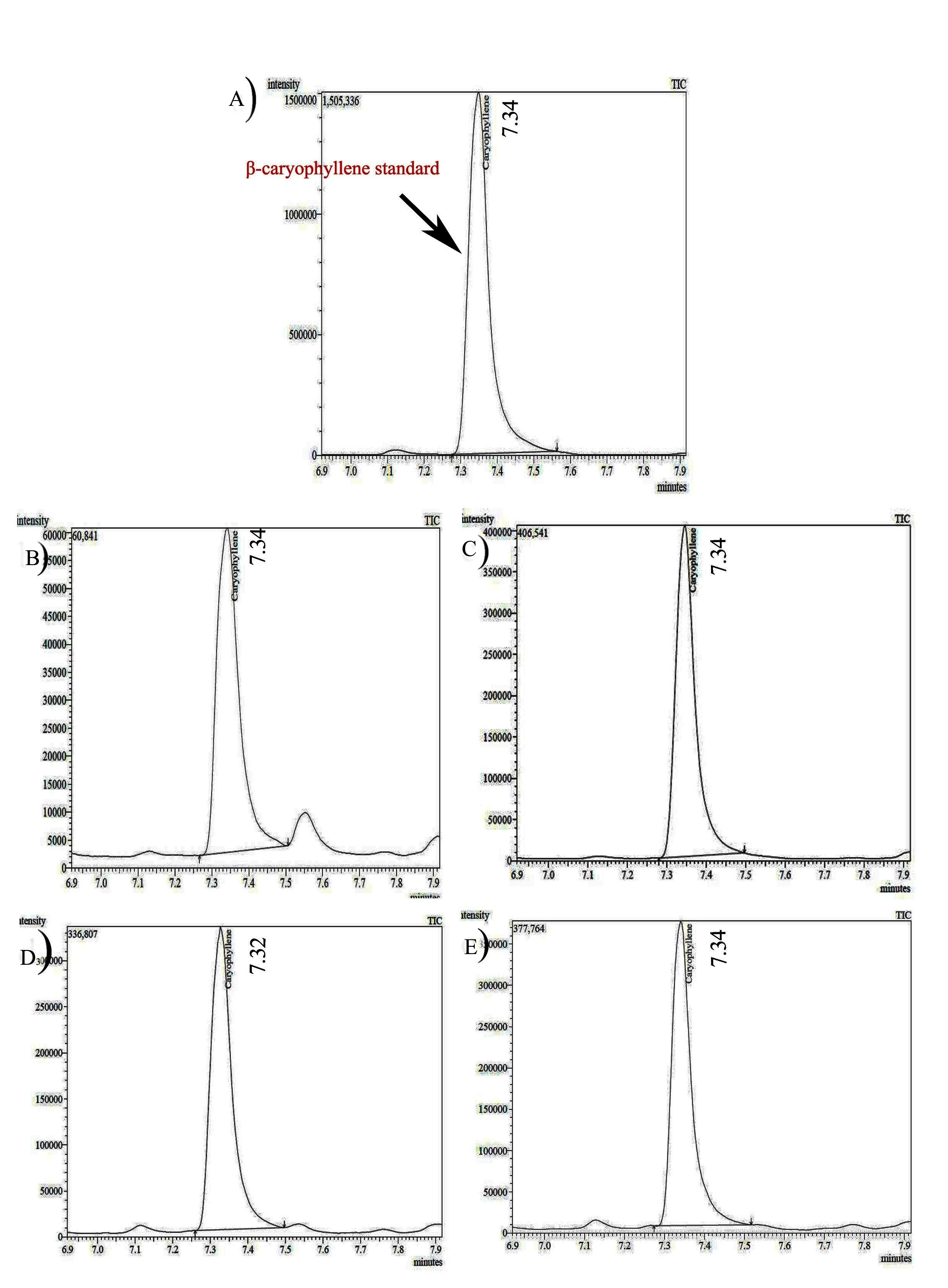


**Figure S1**

| Figure Peaks | R.Time | F.Time | M/z | Area | Height |
| --- | --- | --- | --- | --- | --- |
| A) **standard**  β-caryophyllene | 7.34 | 7.62 | 93.00 | 2206104 | 543068 |
| B) 2,4D 9 µM | 7.34 | 7.50 | 93.00 | 93365 | 21263 |
| C) 2,4D 13.5 µM | 7.32 | 7.50 | 93.00 | 356057 | 118940 |
| D) 2,4D 9 µM + FDP 3 µM | 7.34 | 7.50 | 93.00 | 477722 | 136491 |
| E) 2,4D 13.5 µM + FDP 3 µM | 7.34 | 7.50 | 93.00 | 525317 | 146786 |


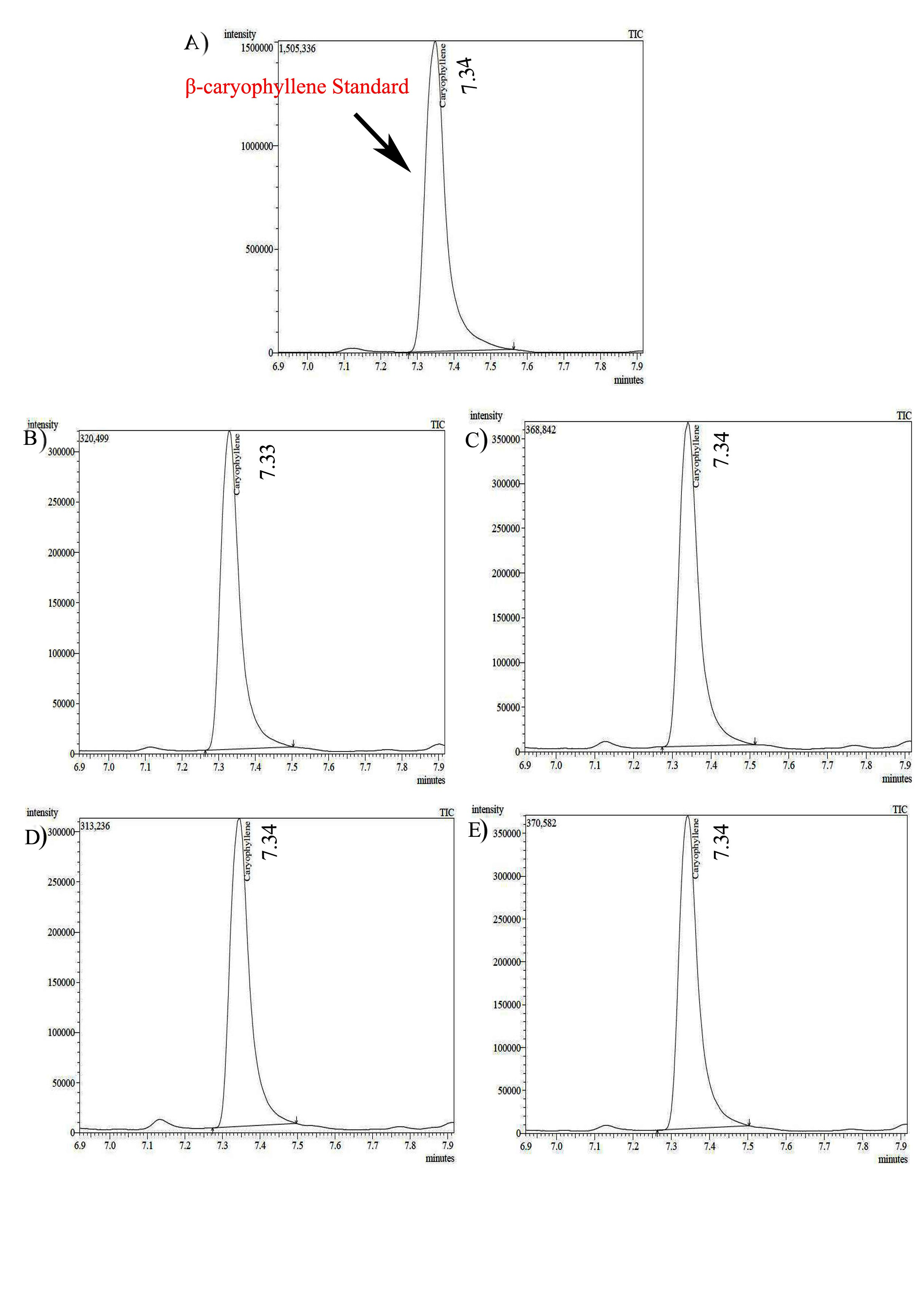


**Figure S2**

| Peak | R.Time | F.Time | M/z | Area | Height |
| --- | --- | --- | --- | --- | --- |
| A) **standard** β-caryophyllene | 7.34 | 7.62 | 93.00 | 2206104 | 543068 |
| B) NAA 10.7 µM | 7.33 | 7.50 | 93.00 | 436007 | 115230 |
| C) NAA 16.1 µM | 7.34 | 7.50 | 93.00 | 492361 | 135213 |
| D) NAA 10.7µM + FDP 3 µM | 7.34 | 7.50 | 93.00 | 491788 | 132208 |
| E) NAA 16.1 µM + FDP 3 µM | 7.34 | 7.50 | 93.00 | 493407 | 132849 |


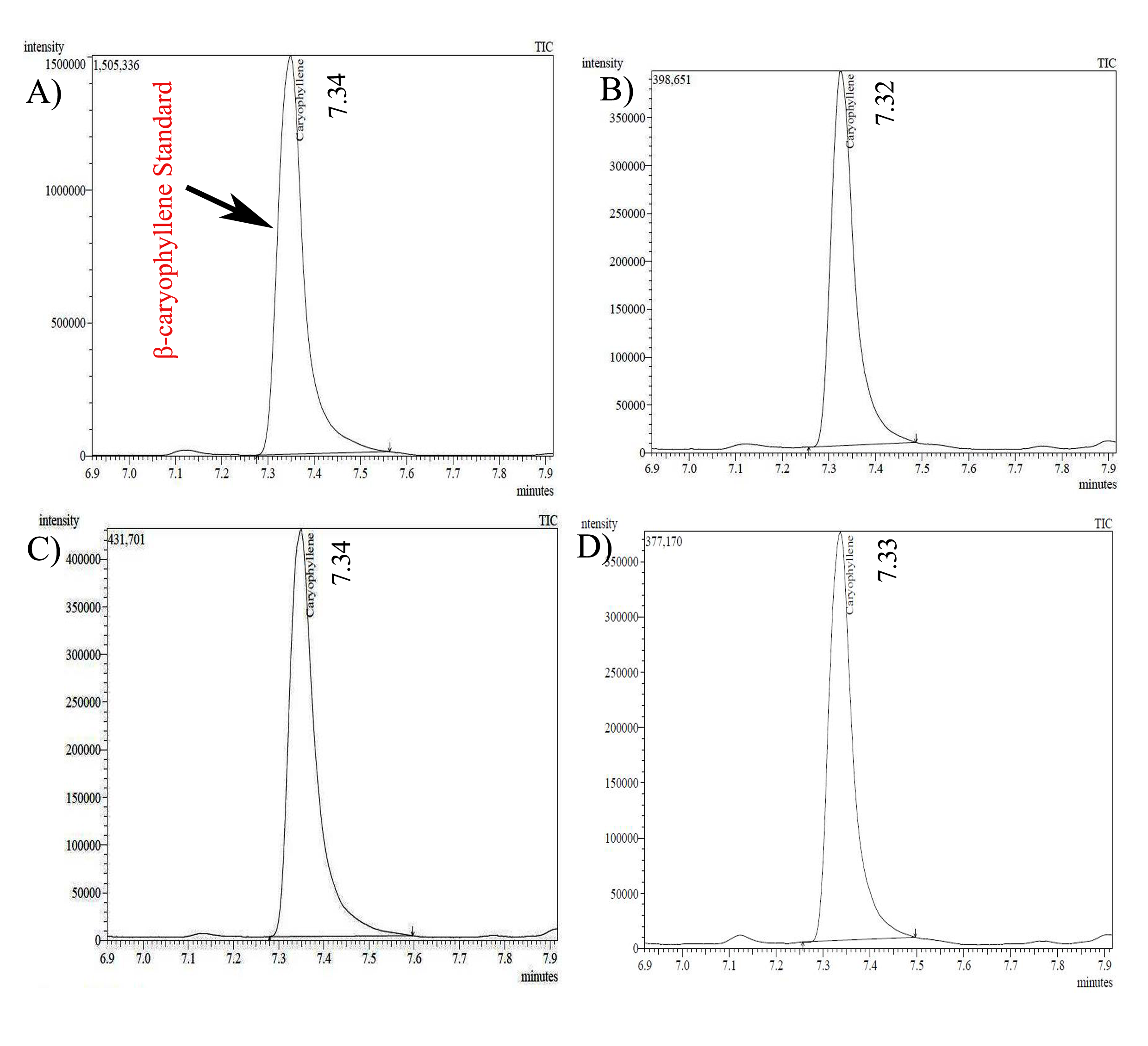


**Figure S3**

| Peak | R.Time | F.Time | M/z | Area | Height |
| --- | --- | --- | --- | --- | --- |
| A) **standard** β-caryophyllene | 7.34 | 7.62 | 93.00 | 2206104 | 543068 |
| B) ½ MS | 7.32 | 7.49 | 93.00 | 538463 | 143646 |
| C) ½ MS + FDP 3 µM | 7.34 | 7.60 | 93.00 | 644370 | 154907 |
| D) ½ MS + B_5_ vitamins + FDP 3 µM | 7.33 | 7.50 | 93.00 | 630420 | 149523 |

**
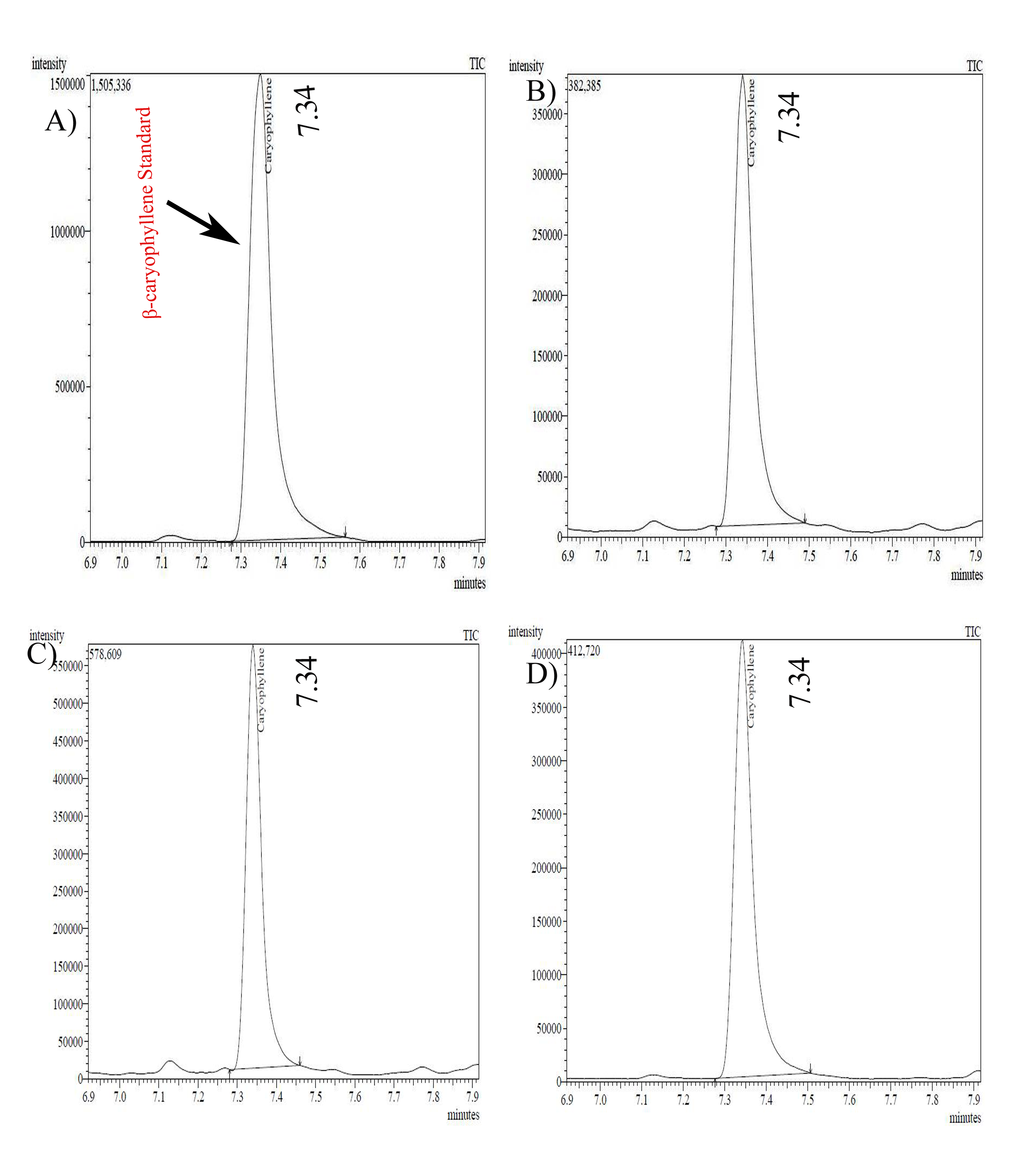
**

**Figure S4**

| Peak | R.Time | F.Time | M/z | Area | Height |
| --- | --- | --- | --- | --- | --- |
| A) **standard** β-caryophyllene | 7.34 | 7.62 | 93.00 | 2206104 | 543068 |
| B) ½ MS | 7.33 | 7.50 | 93.00 | 509825 | 135449 |
| C) ½ MS + FDP 3 µM | 7.34 | 7.45 | 93.00 | 618473 | 207602 |
| D) ½ MS + B5 vitamins + FDP 3 µM | 7.34 | 7.50 | 93.00 | 609224 | 196500 |


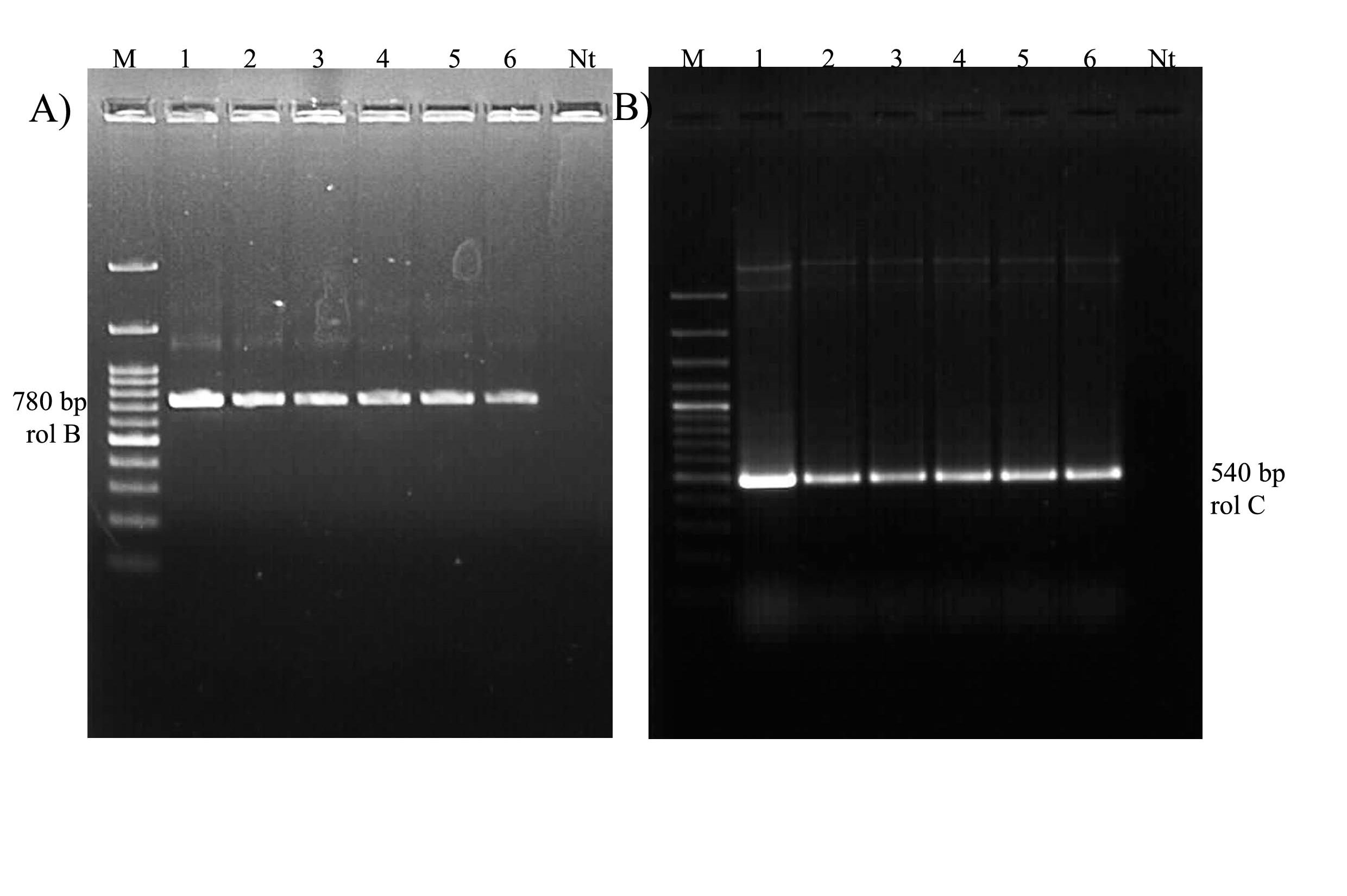


**Figure S5**

**Supplementary Figure Legends**

**Figure S1**  GC-MS chromatogram of enhanced β-caryophyllene compound with and without precursor treated 2,4D callus

**Figure S2** GC-MS chromatogram of enhanced β-caryophyllene compound with and without precursor treated NAA callus

**Figure S3 GC**-MS chromatogram of enhanced β-caryophyllene compound with precursor treated A_4_ strain hairy root culture

**Figure S4** GC-MS chromatogram of enhanced β-caryophyllene compound with precursor treated R_1000_ strain hairy root culture

**Figure S5** A) rol B gene amplification (780 bp), B) rol C gene amplification (540 bp). Lane M: Marker, Lane 1: A_4_ Plasmid DNA, Lane 2: R_1000_ Plasmid DNA, Lane 3 and 4:Genomic DNA of hairy root culture (A_4_ strain), Lane 5 and 6: Genomic DNA of hairy root culture (R_1000_ strain) Nt: Non transgenic root
